# The Impact of Social Isolation on Cognition and Quality of Life Among Stroke Patients in China

**DOI:** 10.1093/geroni/igab046.255

**Published:** 2021-12-17

**Authors:** Xiangjing Kong, Juan Li, Zhijian Liu, Jing Wang, Bei Wu

**Affiliations:** 1 School of Nursing Second Military Medical University, Shanghai, Shanghai, China (People's Republic); 2 Department of clinical nursing School of Nursing Second Military Medical University, Shanghai, Shanghai, China (People's Republic); 3 Fudan University, shanghai, Shanghai, China (People's Republic); 4 New York University, New York, New York, United States

## Abstract

This study examined the impactof social isolation on cognitive function and Quality of Life (QoL) among acute ischemic stroke (AIS) patients in China. We conducted in-person interviews among 206 AIS patients during the acute stage and at 3-month after onset in three cities between May 2020 and February 2021. The data was collected during and post-COVID-19 period in China. We conducted bivariate and multipleregression analyses.Results show that over time, average level of social isolation decreased, and cognitive function and QoL increased.After controllingfor covariates, social isolation was negatively associated with cognitive function (β=-0.438, p<0.01) and QoL (β=-2.521, p<0.01). These findings suggest that addressing the issue of social isolation could potentially impact patients’ cognitive function and QoL.Future studies are needed to further examine the linkages between long-term social isolation and changes in cognitive function and QoL among AIS patients.

